# Phosphodiesterase 5 inhibitors lower both portal and pulmonary pressure in portopulmonary hypertension: a case report

**DOI:** 10.1186/1752-1947-1-46

**Published:** 2007-07-10

**Authors:** Hinrich C Bremer, Wolfgang Kreisel, Kai Roecker, Michael Dreher, Daniel Koenig, Anna Katharina Kurz-Schmieg, Hubert E Blum, Martin Roessle, Peter Deibert

**Affiliations:** 1 Department of Pneumology, University Hospital, Freiburg, Germany; 2 Department of Gastroenterology, Hepatology, Endocrinology and Infectious Diseases, University Hospital, Freiburg, Germany; 3 Department of Rehabilitative and Preventive Sports Medicine, University Hospital, Freiburg, Germany; 4 Gastrointestinal and Endocrinological Center, Freiburg, Germany

## Abstract

**Background:**

Portopulmonary hypertension (PPHTN) is a severe complication in liver cirrhosis. PDE5 inhibitors lower pulmonary arterial pressure (PAP) in PPHTN. However, their effect on portal hypertension has not yet been investigated.

**Case presentation:**

A 55 year old male patient presented with PPHTN and alcoholic liver cirrhosis. 10 mg of Tadalafil, a PDE5 inhibitor with a long half-life, was administered orally under continuous monitoring of pulmonary and portal hemodynamics. For maintenance therapy the patient received Sildenafil 20 mg bid.

Tadalafil lowered mean PAP from 45 to 39 mmHg within 60 minutes. Cardiac output (CO) increased from 6.8 to 7.9 l/min. Central venous pressure (CVP) remained stable at 3 mmHg. Systolic and diastolic blood pressure was lowered from 167/89 to 159/86 mmHg. Pulse rate increased from 75 to 87 per min. Wedged hepatic vein pressure (WHVP) decreased from 21 to 18 mm Hg, hepatovenous pressure gradient (HVPG) decreased from 10 to 7 mmHg. Hemodynamic monitoring after 6 months of Sildenafil therapy revealed a sustained lowering of mean PAP. HVPG remained constant at 10 mmHg. Cardiac and pulmonary performance had further improved.

**Conclusion:**

This case report shows for the first time, that phosphodiesterase 5 inhibitors lower both portal and pulmonary pressure in portopulmonary hypertension.

## Background

Liver cirrhosis may be complicated by the portopulmonary hypertension (PPHTN), a combination of portal hypertension and pulmonary hypertension. A dysregulation of the NO-cGMP system as described for the liver sinusoids in liver cirrhosis [1] may play an important role in PPHTN, too. Cytokines bypassing the liver via collateral blood flow may lead to an imbalance of vasoactive substances in pulmonary vessels, which respond with active vasoconstriction and proliferation of endothelial and smooth-muscle cells.

PPHTN has an estimated prevalence in liver transplant candidates of 3–6%, and in patients with refractory ascites as high as 16% and is the third most frequent type of pulmonary arterial hypertension [2]. The prognosis is poor, with a mean survival of 15 months after diagnosis and even poorer in patients with a mean PAP > 35 mmHg. After liver transplantation the risk of complications is markedly elevated.

Beta-blockers – the standard medical treatment of portal hypertension – worsen the prognosis of patients with PPHTN [3]. Endothelin receptor blockers and prostacyclin derivates used in idiopathic pulmonary hypertension (IPAH) have also been tested in PPHTN with promising results. PDE5 inhibitors, a recently accepted therapy of IPAH [4], have been shown to lower PAP in PPHTN, too. However, their effects on portal pressure have not been investigated so far. We have shown that Vardenafil, an PDE5 inhibitor with a short half-life, lowers portal pressure in healthy subjects and patients with liver cirrhosis [5]. Here we show that Tadalafil, a PDE5 inhibitor with a long half-life, reduces both PAP and portal pressure in a patient with PPHTN. Maintenance therapy with Sildenafil improved the cardio-pulmonary performance without negative effects on portal pressure.

## Case presentation

A 55 year old male patient with Child A alcoholic liver cirrhosis was admitted to the hospital because of increasing dyspnoea at exercise. He had been completely abstinent from alcohol since 7 years. There was no prior history of hemoptysis or gastrointestinal bleeding. The patient reported two episodes of syncope initiated by exercise. He was obese (186 cm, 108 kg). Examination of the heart and the lungs was unremarkable. The liver was enlarged and palpable. No edema was present. The patient could climb 3 flights of stairs (NYHA II). There were no signs of hepatic encephalopathy. The patient received no current medication.

Blood pressure was 140/105 mmHg, heart rate was 79/min. ECG indicated a dilation of right atrium and increased right heart pressure. Doppler echocardiogram showed right heart enlargement with a PAP_systolic _of about 75 mmHg and normal dimensions of the left ventricle and atrium. Holter ECG did not reveal any severe arrhythmias.

Abdominal duplex sonography showed a slow portal blood flow (9 cm/s) and a reduced portal flow volume (0.15 l/min) with intrahepatic retrograde perfusion. The umbilical vein was open and a large splenorenal shunt was detected. Ascites was absent. Second grade esophageal varices were found at endoscopy.

Pathological laboratory findings were thrombocytopenia (112.000/μl), prolonged prothrombin time (68%), elevated bilirubin (3.3 mg/dl), and yGT (60 U/l). Spirometry showed a normal vital capacity (5.35 l, 103% predicted) and FEV1 (3.4 l, 86% predicted), respectively. Endexpiratory flow (MEF 25) was reduced to 0.5 l (22% predicted). Six-minute walking distance was 522 m. Arterial blood gas analysis before and after six-minute walking test showed normal p_a_O_2 _(74 and 75 mmHg) and p_a_CO_2 _(35 and 32 mmHg). After exclusion of other causes of pulmonary hypertension according to current guidelines portopulmonary hypertension was diagnosed in this patient.

**Figure 1:**
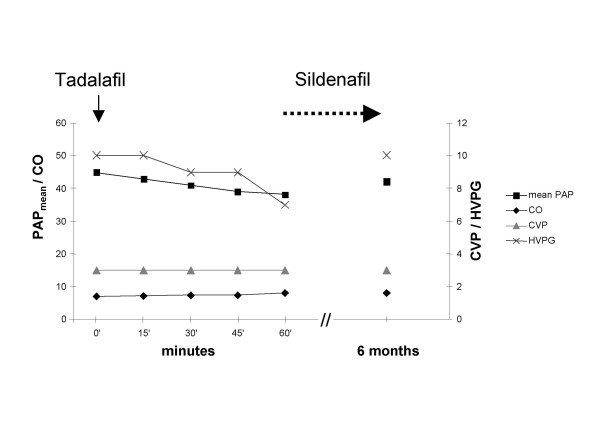
Reduction of mean pulmonary arterial pressure (PAP_mean_; solid square; mmHg) and hepatovenous pressure gradient (HVPG; cross; mmHg), cardiac output (CO; black diamond; l/minm^2^) and central venous pressure (CVP; gray triangle; mmHg) after a single oral Tadalafil administration and after Sildenafil therapy for the following 6 months.

We tested the effect of 10 mg Tadalafil on pulmonary and hepatic hemodynamics invasively in the short term after right heart catheterization and introducing a balloon catheter into an intermediate liver vein. As we already had some experience with Sildenafil and Vardenafil, two other PDE5 inhibitors, in liver cirrhosis, we were interested in the effect of Tadalafil in this case. Tadalafil, the PDE5 inhibitor with a long half-life could be the most suitable substance for long-term therapy. The hemodynamic test was approved by the local ethics committee. Fig. 1 shows the time course of pulmonary and hepatic hemodynamic parameters. After 60 minutes PAP_mean _was reduced from 45 to 39 mmHg. Cardiac output increased from 6.8 to 7.9 l/min and pulmonary vascular resistance decreased from 459 to 344 dyn·sec·cm^-5^. Arterial p_a_O_2 _increased from 70.5 to 78.2 mmHg (Table 1). PAWP was 6 mm Hg, CVP was 3 mm Hg before and 60 minutes after Tadalafil. Wedged hepatic vein pressure (WHVP) decreased from 21 to 18 mmHg, free hepatic vein pressure (FHVP) remained constant at 11 mmHg. HVPG (WHVP – FHVP) decreased by 30%.

**Table 1:** Hemodynamic and pulmonary parameters

		After 3 months Sildenafil		After 6 months Sildenafil		
Parameter	Baseline	After 60 minutes Tadalafil	60 min after the last dose	8 h after the last dose	60 min after 20 mg Sildenafil
Systolic BP (mmHg)	167	159	147	145	140
Diastolic BP (mmHg)	89	86	97	100	90
Heart rate (1/min)	75	87	93	69	75
Cardiac index (l/minm^2^)	3.02	3.51		3.33	3.56
Cardiac output (l/min)	6.8	7.9		7.5	8.0
Mean PAP (mmHg)	45	39		42	42
PVR (dyn·s^-1^·cm^-5^)	459	344		352	360
Central venous pressure (mmHg)	3	3		6	3
PAWP	6	6		9	6
Hepatovenous pressure gradient (mmHg)	10	7		10	
Arterial p_a_O_2 _(mmHg)	70.5	78.2	71.4	76.9	
Arterial p_a_CO_2 _(mmHg)	41.3	38.4	35.3	31.3	
6 min walking test (m)	522		514	580	
VO_2peak _(ml/min)	2020	2240		2640	
Watt max	120	130		140	
VE/VO_2_	37.0	33.5		29.3	
VE/VCO_2_	35.2	33.2		27.8	

Hemodynamic and pulmonary parameters after inhibition of PDE5. BP = blood pressure, PAP = pulmonary arterial pressure, PVR = pulmonary vascular resistance, VO_2peak _= peak oxygen uptake, VE/VO_2 _ventilatory equivalent of oxygen; VE/VCO_2 _ventilatory equivalent of carbon dioxide. PAWP = pulmonary arterial wedged pressure

One week later the effect of Tadalafil on exercise capacity was investigated (Table 1). Breath gas analysis with cycling ergometry up to subjective exhaustion (120 W) at baseline showed a VO_2peak _of 2020 ml/min. At maximum exhaustion, VE/VCO_2 _was 35.2 and VE/VO_2 _was 37.0, respectively. Cycling ergometry was repeated on the following day one hour after Tadalafil administration. VO_2peak _during cycling exercise was slightly increased (2240 ml/min, 130 W) with a consistent decrease in VE/VCO_2 _(33.2) and VE/VO_2 _(33.5) at 130 W. These decreased ventilatory equivalent ratios (ventilation (VE) divided by oxygen consumption (VO_2_) or carbon dioxide release (VCO_2_)) reflect better ventilatory efficiency.

Thereafter, the patient received Sildenafil, since this PDE5 inhibitors is approved for treatment of IPAH. The dose was reduced to 20 mg bid as compared to the dose used in recent studies considering the altered pharmacology of the drug in liver cirrhosis. Clinical symptoms and laboratory tests were recorded in our outpatient clinic. No adverse side effects were seen. Six minutes walking test after three months was unchanged, but was increased to 580 m after 6 months.

Six months after starting therapy with Sildenafil exercise capacity and pulmonary hemodynamics were measured again. Breath gas analysis during cycling exercise resulted in an increased VO_2peak _(2640 ml/min, 140 W). A further decrease in VE/VCO_2 _(27.8) and VE/VO_2 _(29.9) confirmed an improved cardiopulmonary function.

8 hours after the morning Sildenafil dose hemodynamic parameters were invasively measured before and 60 minutes after oral intake of 20 mg Sildenafil. After 60 minutes PAP_mean _remained unchanged at 42 mmHg. Cardiac output increased from 7.5 to 8.0 l/min, pulmonary vascular resistance remained unchanged at 352 and 360 dyn·sec·cm^-5^, respectively. HVPG before Sildenafil was 10 mmHg (WHVP 21 mmHg, FHVP 11 mmHg). After 60 minutes the liver catheter was occluded by a small thrombus and WHVP and FHVP could not be determined.

## Discussion and conclusion

Here we describe the reduction of both pulmonary arterial and portal venous pressure in a patient with portopulmonary hypertension by PDE5 inhibitors with a beneficial effect on cardiac and pulmonary performance. Tadalafil – a long acting PDE5 inhibitor – lowered PAP_mean _and pulmonary arterial resistance within 60 minutes. There are several reports on the effect of Sildenafil on PAP_mean _in PPHTN [6]. However, the effect of a PDE5 inhibitor on portal pressure in PPHTN has not yet been evaluated. The data from the present patient confirms our previous results [5]: In this study we had shown that in liver cirrhosis the PDE5 inhibitor Vardenafil induced a rapid increase of portal venous blood flow by about 20–25% and a decline of HVPG by about 20% in liver cirrhosis.

Wang et al. [7] reported on an increase of WHVP and HVPG in a patient with PPHTN after Sildenafil accompanied by an increase of arterial blood pressure and heart rate. The latter effects are highly atypical for Sildenafil, so one may speculate that other effects may have interfered with the genuine effects of Sildenafil on portal pressure. There are case reports about a correlation between Sildenafil intake and bleeding from esophageal varices. It was speculated that Sildenafil may increase portal pressure. However, these reports did not differentiate between bleeding caused by sexual activity after Sildenafil intake and a hypothetical deleterious effect of the drug on portal pressure [8,9]. This case presentation and recent own results [5] suggest that PDE5 inhibitors are no major risk factor for bleeding from esophageal varices.

Data from animal experiments showed inconsistent results for the effect of Sildenafil on portal flow and portal pressure [10]. However, these data cannot be transferred to the clinical setting, since Sildenafil was administered intravenously or intraarterially as a bolus and at excessively high doses resulting in drug levels several orders of magnitude higher than after therapeutic oral application in humans.

In our patient, 6 months treatment with Sildenafil induced a marked improvement of exercise capacity. The hemodynamic follow-up measurements after 6 months therapy showed no additional effect of Sildenafil on PAP_mean _and pulmonary vascular resistance 8 hours after the last dose. Probably even trough levels are sufficient to exert the maximum dilating effect on the pulmonary vessels. It may be speculated that PDE5 inhibition relieved the vasoconstriction in the acute testing, but that a further vasodilating effect could not be expected on the long term. Unfortunately, the effect of Sildenafil in this situation could not be determined due to technical problems. However, a deterioration of portal hemodynamics can be excluded.

Beta-blockers for prevention of bleeding are contraindicated in patients with PPHTN, since they worsen the pulmonary hemodynamics by pulmonary vasoconstriction and limitation of right ventricular function [3]. In analogy to other forms of pulmonary arterial hypertension therapy with PDE5 inhibitors is used in PPHTN. Our data add a further rationale for application of these drugs since a dual effect may be achieved, lowering of pulmonary and portal pressure. The biochemical background for regulation of sinusoidal tonus [1] suggests that PDE5 inhibitors act by at least partially reversing the sinusoidal constriction that is characteristic for liver cirrhosis. Our data suggest that Sildenafil, Vardenafil, and Tadalafil, have comparable effects on portal hemodynamics: oral administration of the drugs at doses, which are used for erectile dysfunction, improves portal liver perfusion and induces a drop of portal pressure or HVPG as is required for effective prevention of bleeding. Therefore, the potential beneficial action of PDE5 inhibitors in liver cirrhosis should be evaluated in clinical studies.

## Abbreviations

CO cardiac output

CO_2 _carbon dioxide

cGMP cyclic guanosine monophosphate

CVP central venous pressure

eNOS endothelial NO-synthase

FHVP free hepatic venous pressure

HVPG hepato-venous pressure gradient

IPAH idiopathic pulmonary arterial hypertension

NO nitric oxide

WHVP wedged hepatic vein pressure

PAWP pulmonary arterial wedged pressure

PAP pulmonary arterial pressure

PAP_mean _mean pulmonary arterial pressure

PAP_systolic _systolic pulmonary arterial pressure

PDE5 phosphodiesterase 5

PPHTN portopulmonary hypertension

O_2 _oxygen

yGT gamma-glutamyltransferase

VE ventilatory equivalent

VO_2peak _peak oxygen uptake

## Competing interests

The author(s) declare that they have no competing interests.

## Authors' contributions

PD and WK had the idea to use PDE5 inhibitors for lowering portal pressure. WK, PD, HB, MR and MD did the invasive measurements. Spiroergometry was performed by KR. Duplex sonography was done by PD and AKKS. DK and HEB contributed to the writing of the paper. All authors read and approved the final manuscript.
